# Percutaneous coronary intervention and 30‐day unplanned readmission with chest pain in the United States (Nationwide Readmissions Database)

**DOI:** 10.1002/clc.23543

**Published:** 2021-02-16

**Authors:** RobertA. Sykes, Mohamed O. Mohamed, Chun Shing Kwok, Mamas A. Mamas, Colin Berry

**Affiliations:** ^1^ Institute of Cardiovascular and Medical Sciences University of Glasgow Glasgow UK; ^2^ Golden Jubilee National Hospital UK; ^3^ Keele University Keele Staffordshire UK; ^4^ Royal Stoke University Hospital UK

**Keywords:** acute coronary syndromes, angina, chest pain, chronic coronary syndromes, ischemic, percutaneous coronary intervention, readmissions, heart disease, outcomes

## Abstract

Percutaneous coronary intervention (PCI) improves anginal chest pain in most, but not all, treated patients. PCI is associated with unplanned readmission for angina and non‐specific chest pain within 30‐days of index PCI. Patients with an index hospitalization for PCI between January–November in each of the years 2010–2014 were included from the United States Nationwide Readmissions Database. Of 2 723 455 included patients, the 30‐day unplanned readmission rate was 7.2% (*n* = 196 581, 42.3% female). This included 9.8% (*n* = 19 183) with angina and 11.1% (*n* = 21 714) with non‐specific chest pain. The unplanned readmission group were younger (62.2 vs 65.1 years; *P* < 0.001), more likely to be females (41.0% vs 34.2%; *P* < 0.001), from the lowest quartile of household income (32.9% vs 31.2%; *P* < 0.001), have higher prevalence of cardiovascular risk factors or have index PCI performed for non‐acute coronary syndromes (ACS) (OR:3.46, 95%CI 3.39–3.54). Factors associated with angina readmissions included female sex (OR:1.28, 95%CI 1.25–1.32), history of ischemic heart disease (IHD) (OR:3.28, 95%CI 2.95–3.66), coronary artery bypass grafts (OR:1.79, 95%CI 1.72–2.86), anaemia (OR:1.16, 95%CI 1.11–1.21), hypertension (OR:1.13, 95%CI 1.09, 1.17), and dyslipidemia (OR:1.10, 95%CI 1.06–1.14). Non‐specific chest pain compared with angina readmissions were younger (mean difference 1.25 years, 95% CI 0.99, 1.50), more likely to be females (RR:1.13, 95%CI 1.10, 1.15) and have undergone PCI for non‐ACS (RR:2.17, 95%CI 2.13, 2.21). Indications for PCI other than ACS have a greater likelihood of readmission with angina or non‐specific chest pain at 30‐days. Readmissions are more common in patients with modifiable risk factors, previous history of IHD and anaemia.

## Graphical abstract

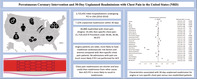

## 1 INTRODUCTION

Percutaneous coronary intervention (PCI) is indicated for acute coronary syndromes (ACS) or the relief of anginal symptoms secondary to myocardial ischemia, in patients with obstructive coronary artery disease (CAD). Around 3 million procedures are performed worldwide every year. The results of recent randomized, controlled trials of clinical strategies involving invasive management of CAD have not provided evidence of clear benefits for coronary revascularization over medical therapy in patients with chronic coronary syndromes (CCS).^1^, ^2^

In the ABSORB‐4 trial, which compared clinical outcomes in patients treated with either a bioresorbable scaffold or a 3rd generation drug eluting stent, the occurrence and time‐course of angina post‐PCI was similar in both groups, occurring in 11% of subjects by 30 days and 22% of patients by 1‐year.^3^ The clinical characteristics associated with anginal chest pain at these time‐points and experience in less‐selected, real‐world populations are uncertain. Readmission within 30 days following PCI is not uncommon, with a broad spectrum of etiologies and degrees of severity.^4^ Readmissions are commonly secondary to cardiac‐related disorders or PCI complications and it is reported that readmission is associated with a greater risk of mortality.^5^, ^6^, ^7^, ^8^

In this study, we accessed a large, national readmissions database to investigate the proportion of patients re‐admitted to hospital with chest pain attributed to angina or non‐specific chest pain within 30 days after PCI for ACS or CCS and the associated clinical characteristics. In addition, we evaluated the cost burden of chest pain readmissions compared with readmissions due to other causes.

## 2 METHODS

In the United States, the Healthcare Cost and Utilization Project (HCUP) Nationwide Readmissions Database (NRD) records, hospitalization and readmission data for all hospitalized patients within 21 States and is produced by the Agency for Healthcare Research and Quality. This study utilized deidentified data collected and distributed by HCUP and does not require consent from individual patients or an institutional board review (IRB) approval. The distribution of included municipalities is geographically diverse and represents 49.1% of all hospital inpatients, including patients with and without insurance. Admissions are linked by an individual identification number, which enables linkage between admissions independent of readmission location.

Patients were included if they are 18 years or older and underwent PCI at index admission (ICD‐9 Procedure code: 00.66, 36.06, 36.07) with discharge data from 2010 to 2014. Only the first admission with PCI within a calendar year was considered. Cases were excluded if they died during index admission, had duplicate data, were missing demographic or readmission data, or readmitted electively. Additionally, patients admitted in December are excluded as they lack 30 days of follow‐up. Patients who were readmitted with a primary chest pain diagnosis are defined by ICD‐9 codes (*Angina* – ICD‐9: 413.0, 413.1, 413.9; non‐specific chest pain [*NSCP*] – ICD‐9: 786.5, 786.51) and clinic classification software codes (*stable coronary artery disease including angina* – CCS: 101; *Non‐specific chest pain* – CCS: 102; see Table A1). Demographic, comorbidity at index admission and outcome data as well as detail of inpatient stay was captured through a combination of NRD coding, ICD‐9 and Elixhauser comorbidity codes. Cost‐to‐charge ratios were applied to total charges as recommended by HCUP in order to provide an estimate of inpatient cost.

The primary outcome of this analysis is 30‐day readmission with a primary diagnosis of chest pain post‐PCI, and variables associated with readmission. A sub‐group analysis of the characteristics of patients with a primary diagnosis of angina and non‐specific chest pain is also performed.

Statistical analysis was performed using IBM Statistics SPSS (version 24.0). Weighting is performed using sample discharge weights. Dichotomization of patients based on the presence or absence of readmission within 30 days and subsequent descriptive statistics are presented. Chi‐square or Independent Student‐T testing with 95% two‐tailed significance was utilized for comparing patient demographics. Multiple logistic regression analyses were performed to evaluate the association between these variables and readmission within 30‐days with angina, non‐specific chest pain and the combined population readmitted with angina or non‐specific chest pain. Furthermore, the relative risk (RR) of association with variables and readmission within 30‐days of angina versus non‐specific chest pain is also evaluated.

## 3 RESULTS

**FIGURE 1:**
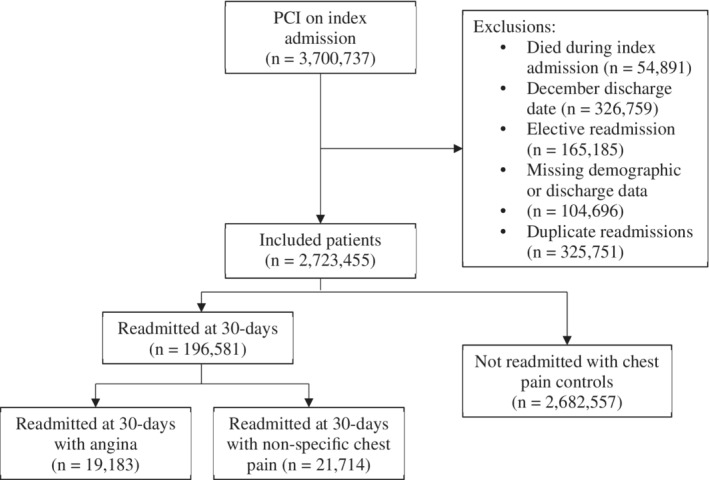
Flow diagram

Of 3 700 737 identified as undergoing PCI in the United States in the years 2010–2014, 2 723 455 were included in the analysis. The reasons for exclusion are described in Figure 1. Of note, 326 759 were excluded due to a December discharge date. In total, 104 696 patients were excluded from analysis due to missing demographic, discharge or mortality data.

### 3.1 Characteristics of patients readmitted for chest pain or angina within 30‐days post‐PCI

**FIGURE 2:**
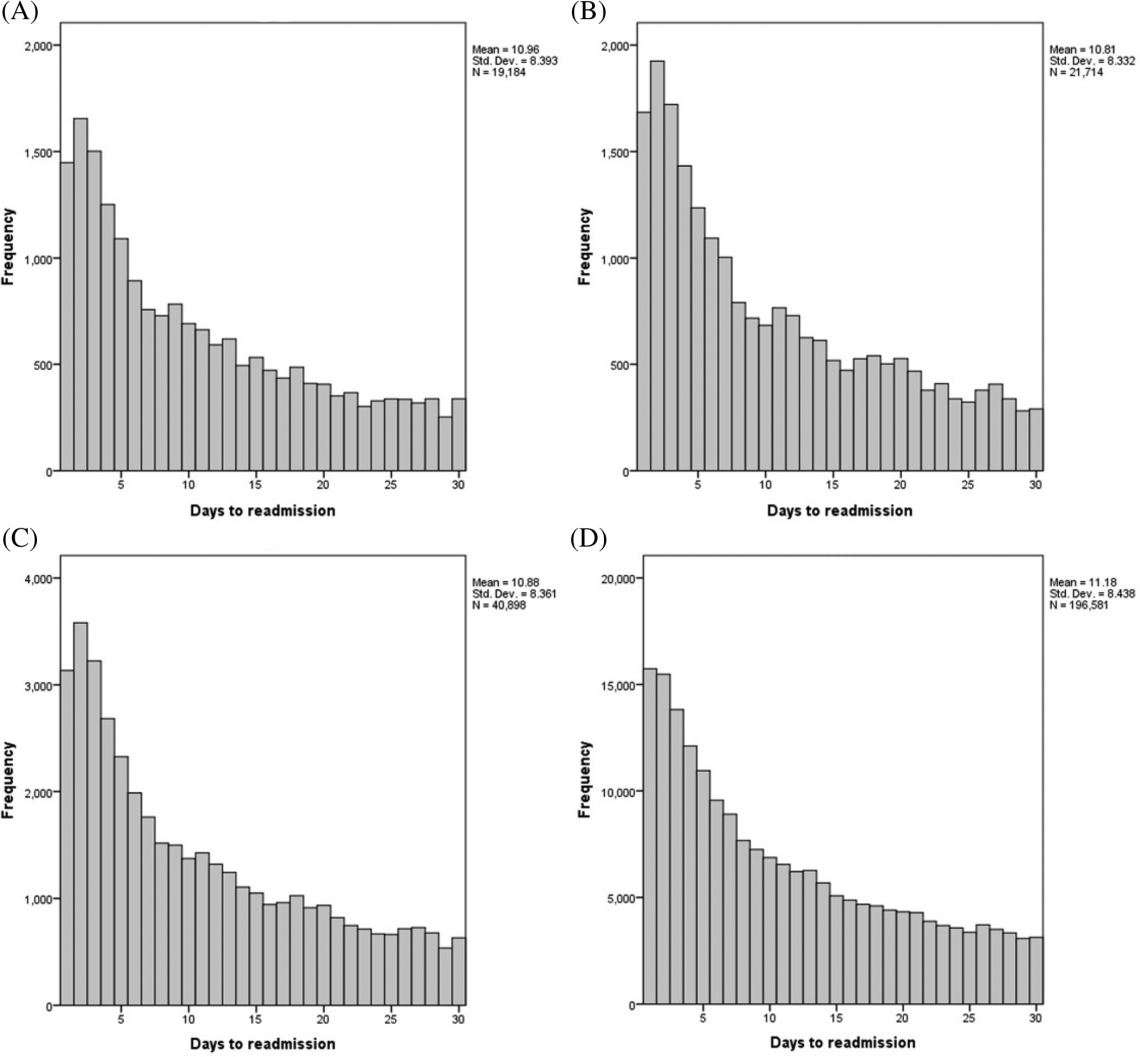
Distribution of readmissions with angina (A), non‐specific chest pain (B), all chest pain (C), all‐causes of readmission (D) within 30‐days (*X axis: days to readmission; Y axis: frequency*)

Within this post‐PCI cohort (*n* = 2 723 455), 196 581 (7.2%) had an unplanned hospital readmission for any cause within 30‐days. Of these, 40 897 (20.8%) patients (1.5% of the whole post‐PCI cohort) were readmitted with a primary diagnosis of chest pain at 30 days, including 19 183 patients with angina and 21 714 with non‐specific chest pain. The demographics and medical history of the patients who were or were not readmitted during a 30‐day period are provided within Table 1. Peak readmissions for chest pain, angina and non‐specific chest pain are observed within the first 48 h following discharge post‐PCI (Figure 2).

**TABLE 1:** Patient demographics and characteristics including co‐morbidities at index admission

Variable	All patients				All chest pain		NSCP		Angina	
			Control group No readmission		Unplanned readmission		Unplanned readmission		Unplanned readmission	
	(*n* = 2 723 455)		(*n* = 2 682 557)		(*n* = 40 897)		(*n* = 21 714)		(*n* = 19 183)	
Age in years (SE)	65.1	(0.01)	65.1	(0.01)	62.2	(0.07)	61.6	(0.09)	62.8	(0.10)
Female	934 574	34.3%	917 814	34.2%	16 760	41.0%	9448	43.5%	7312	38.1%
Length of stay in days (SE)	3.85	(0.00)	3.87	(0.00)	2.39	(0.01)	1.84	(0.01)	3.02	(0.03)
Cost of inpatient stay (SE)	$19 937	(10.6)	$20 133	(10.7)	$7083	(48.7)	$5064	(24.7)	$9368	(97.3)
Quartile of median household income										
0‐25th	850 333	31.2%	836 868	31.2%	13 465	32.9%	7030	32.4%	6435	33.5%
26th‐50th	721 854	26.5%	711 178	26.5%	10 677	26.1%	5700	26.3%	4977	25.9%
51st‐75th	632 303	23.2%	622 988	23.2%	9315	22.8%	5098	23.5%	4217	22.0%
76th‐100th	518 964	19.1%	511 523	19.1%	7441	18.2%	3886	17.9%	3555	18.5%
Smoker	1 106 460	40.6%	1 091 323	40.7%	15 138	37.0%	7875	36.3%	7263	37.9%
Obesity	421 971	15.5%	416 402	15.5%	5569	13.6%	2917	13.4%	2652	13.8%
Chronic renal failure	407 294	15.0%	401 715	15.0%	5579	13.6%	2738	12.6%	2841	14.8%
Family history of IHD	296 638	10.9%	293 374	10.9%	3264	8.0%	1619	7.5%	1645	8.6%
Personal history of IHD	2 565 060	94.2%	2 526 131	94.2%	38 930	95.2%	20 086	92.5%	18 844	98.2%
Previous MI	457 335	16.8%	447 478	16.7%	9856	24.1%	5233	24.1%	4623	24.1%
Dyslipidemia	1 915 626	70.3%	1 886 214	70.3%	29 412	71.9%	15 301	70.5%	14 111	73.6%
Hypertension	2 032 126	74.6%	2 000 647	74.6%	31 479	77.0%	16 586	76.4%	14 893	77.6%
Diabetes mellitus	1 027 612	37.7%	1 011 495	37.7%	16 118	39.4%	8490	39.1%	7628	39.8%
Heart failure	101 593	3.7%	101 241	3.8%	353	0.9%	76	0.4%	277	1.4%
Valvular heart disease	30 923	1.1%	30 815	1.1%	108	0.3%	18	0.1%	90	0.5%
History of stroke/TIA	161 953	5.9%	159 305	5.9%	2648	6.5%	1451	6.7%	1197	6.2%
Peripheral vascular disease	316 558	11.6%	312 850	11.7%	3708	9.1%	1702	7.8%	2006	10.5%
Anaemia	345 133	12.7%	340 076	12.7%	5058	12.4%	2424	11.2%	2634	13.7%
Atrial fibrillation	349 320	12.8%	345 303	12.9%	4017	9.8%	1799	8.3%	2218	11.6%
Previous CABG	251 465	9.2%	245 327	9.1%	6138	15.0%	3113	14.3%	3025	15.8%
Non‐ACS index PCI	913 026	33.5%	887 497	33.1%	25 529	62.4%	17 208	79.2%	8321	43.4%
ACS index PCI	1 810 429	66.5%	1 795 059	66.9%	15 369	37.6%	4506	20.8%	10 863	56.6%
STEMI	565 264	20.8%	561 394	20.9%	3869	9.5%	1733	8.0%	2136	11.1%
NSTEMI/Unstable angina	1 261 856	46.3%	1 249 663	46.6%	12 193	29.8%	2791	12.9%	9402	49.0%

Abbreviations: ACS, acute coronary syndromes; CABG, coronary artery bypass grafts; IHD, ischemic heart disease; NSCP, non‐specific chest pain; PCI, percutaneous coronary intervention.

Multiple logistic regression models were created to examine the associations between clinical characteristics and co‐morbidities and the likelihood of readmission with a primary diagnosis of angina or non‐specific chest pain at 30 days after PCI. We found several characteristics that were strongly associated with readmission with chest pain at 30‐days (Table 2). The unplanned readmission group were younger (62.2 vs 65.1 years; *P* < 0.001), more likely to be females (41.0% vs 34.2%; *P* < 0.001) and within the lowest quartile of household income (32.9% vs 31.2%; *P* < 0.001). The readmission group also had a higher prevalence of previous ischemic heart disease (IHD), coronary artery bypass grafts (CABG), hypertension and dyslipidemia. Furthermore, they were more likely to have index PCI performed for non‐ACS (odds ratio [OR]: 3.46, 95% CI 3.39, 3.54) (Supplementary Figure 1).

**TABLE 2:** Demographic and clinical characteristics associated with the likelihood of readmission at 30‐days with chest pain following PCI

Variable	All chest pain		NSCP		Angina	
	(*n* = 40 897)		(*n* = 21 714)		(*n* = 19 183)	
	Odds Ratio (95% C.I.)	*P* value	Odds Ratio (95% C.I.)	*P* value	Odds Ratio (95% C.I.)	*P* value
Female	1.44 (1.41, 1.47)	<0.0005	1.57 (1.53, 1.62)	<0.0005	1.28 (1.25, 1.32)	<0.0005
Age	0.97 (0.97, 0.97)	<0.0005	0.97 (0.97, 0.97)	<0.0005	0.98 (0.98, 0.98)	<0.0005
Smoker	0.87 (0.85, 0.89)	<0.0005	0.91 (0.89, 0.94)	<0.0005	0.84 (0.81, 0.86)	<0.0005
Obesity	0.78 (0.76, 0.80)	<0.0005	0.79 (0.76, 0.82)	<0.0005	0.78 (0.75, 0.81)	<0.0005
Q1 (0‐25th %)	1.04 (1.01, 1.07)	0.02	1.01 (0.97, 1.05)	0.57	1.06 (1.02, 1.11)	0.006
Q2 (26‐50th %)	1.00 (0.97, 1.04(	0.787	1.02 (0.98, 1.06)	0.41	0.99 (0.95, 1.03)	0.653
Q3 (51‐75th %)	1.01 (0.98, 1.04)	0.746	1.05 (1.01, 1.10)	0.024	0.96 (0.92, 1.00)	0.06
Q4 (76‐100th %)	0.97 (0.94, 0.99)	0.02	0.99 (0.95, 1.03)	0.572	0.94 (0.91, 0.98)	0.006
Chronic kidney disease	0.94 (0.91, 0.97)	<0.0005	0.88 (0.84, 0.92)	<0.0005	1.00 (0.96, 1.04)	0.916
Family history of IHD	0.74 (0.71, 0.76)	<0.0005	0.74 (0.70, 0.78)	<0.0005	0.74 (0.71, 0.78)	<0.0005
Personal history of IHD	1.20 (1.15, 1.26)	<0.0005	0.76 (0.70, 0.78)	<0.0005	3.28 (2.95, 3.66)	<0.0005
Previous MI	1.36 (1.33, 1.39)	<0.0005	1.28 (1.24, 1.32)	<0.0005	1.44 (1.39, 1.49)	<0.0005
Dyslipidemia	1.08 (1.06, 1.11)	<0.0005	1.07 (1.04, 1.10)	<0.0005	1.10 (1.06, 1.14)	<0.0005
Hypertension	1.12 (1.09, 1.15)	<0.0005	1.11 (1.07, 1.14)	<0.0005	1.13 (1.09, 1.17)	<0.0005
Diabetes Mellitus	0.97 (0.95, 0.99)	0.009	0.96 (0.93, 0.98)	0.002	0.99 (0.96, 1.02)	0.624
Heart Failure	0.18 (0.17, 0.20)	<0.0005	0.07 (0.05, 0.08)	<0.0005	0.36 (0.32, 0.41)	<0.0005
Valvular Heart Disease	0.28 (0.23, 0.34)	<0.0005	0.09 (0.06, 0.14)	<0.0005	0.53 (0.43, 0.65)	<0.0005
History of Stroke/TIA	1.11 (1.07, 1.16)	<0.0005	1.18 (1.11, 1.24)	<0.0005	1.03 (0.97, 1.10)	0.289
Peripheral Vascular Disease	0.76 (0.73, 0.78)	<0.0005	0.65 (0.62, 0.68)	<0.0005	0.88 (0.84, 0.92)	<0.0005
Anaemia	1.04 (1.01, 1.08)	0.009	0.94 (0.89, 0.98)	0.003	1.16 (1.11, 1.21)	<0.0005
Atrial Fibrillation	0.84 (0.82, 0.87)	<0.0005	0.70 (0.66, 0.73)	<0.0005	1.01 (0.97, 1.06)	0.663
Previous CABG	1.67 (1.63, 1.72)	<0.0005	1.54 (1.49, 1.61)	<0.0005	1.79 (1.72, 1.86)	<0.0005
Non‐ACS PCI on index adm.	3.46 (3.39, 3.54)	<0.0005	8.26 (7.99, 8.54)	<0.0005	1.49 (1.45, 1.54)	<0.0005
ACS PCI on index admission	0.29 (0.28, 0.30)	<0.0005	0.12 (0.12, 0.13)	<0.0005	0.67 (0.65, 0.69)	<0.0005

Abbreviations: ACS, acute coronary syndromes; CABG, coronary artery bypass grafts; IHD, ischemic heart disease; NSCP, non‐specific chest pain; PCI, percutaneous coronary intervention.

### 3.2 Characteristics of patients readmitted for angina within 30‐days post‐PCI

**FIGURE 3:**
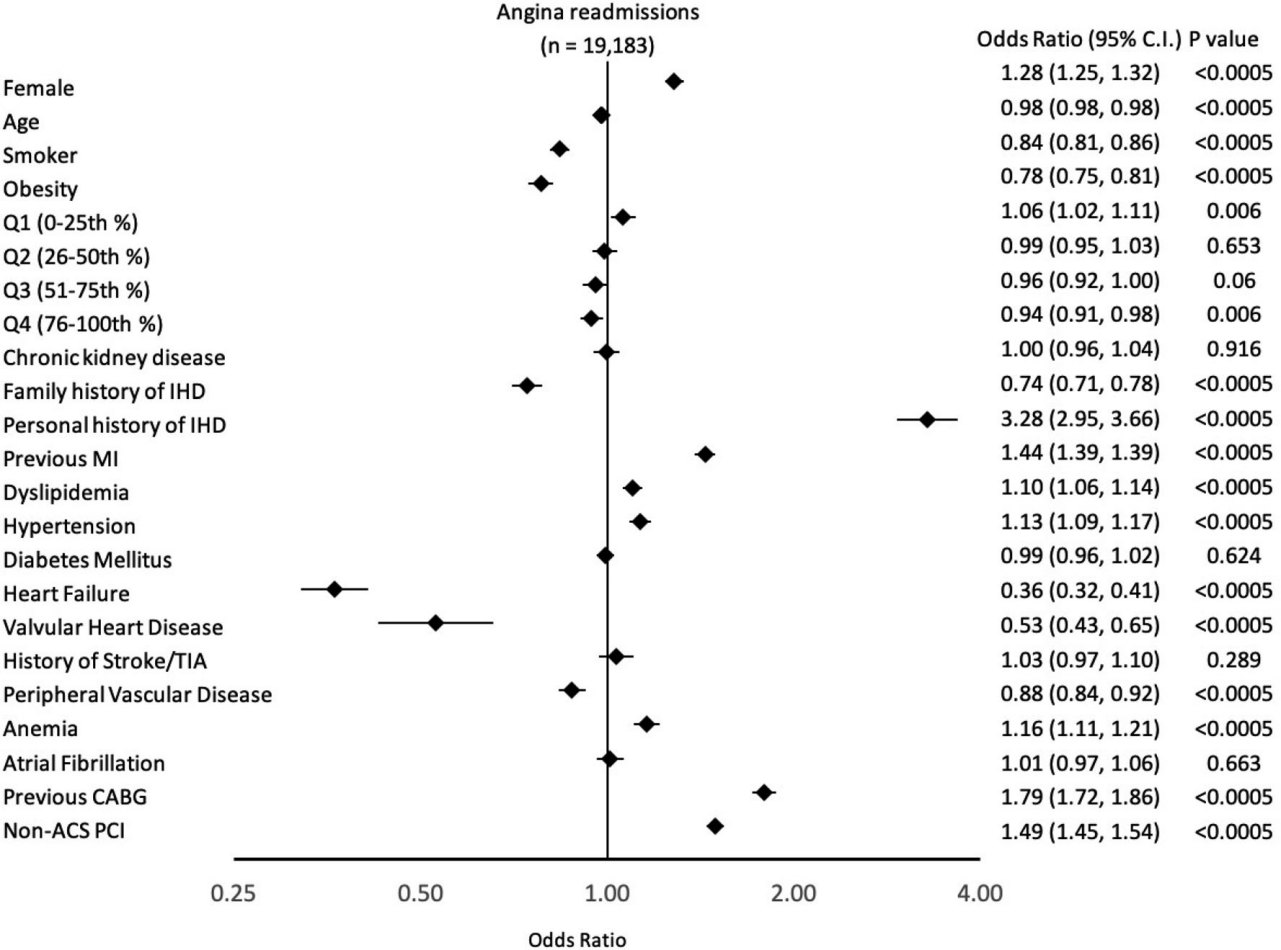
Demographic and clinical characteristics associated with the likelihood of readmission at 30‐days with angina following PCI. PCI, Percutaneous coronary intervention

Patients readmitted with angina within 30‐days were more likely to be female (OR: 1.28, 95% CI 1.25, 1.32), younger (OR: 0.98, 95% CI 0.98, 0.98), associate with the 0–25% of median household income (OR: 1.06, 95% CI 1.02, 1.11), have history of IHD including previous myocardial infarction (OR: 1.44, 95% CI 1.39, 1.49) or coronary artery bypass grafting (OR: 1.79 95% CI 1.72, 1.86, dyslipidemia (OR: 1.10, 95% CI 1.02, 1.14), hypertension (OR: 1.13, 95% CI 1.09, 1.17) and anaemia (OR: 1.16, 95% CI 1.11, 1.21). These patients were also more likely to have undergone index PCI for indications other than ACS (OR: 1.49, 95% CI 1.45, 1.54). Smoking status at index admission, obesity, association with 76–100% of median household income, history of heart failure, valvular heart disease, peripheral vascular disease, and acute coronary syndrome on index PCI (OR = 0.67, 95% CI 0.65, 0.69) were less likely to be observed compared with those who were not readmitted within 30‐days (Figure 3).

### 3.3 Characteristics of patients readmitted for non‐specific chest pain within 30‐days post‐PCI

Of those readmitted with non‐specific chest pain within 30‐days, female gender (OR: 1.57, 95% CI 1.53, 1.62), younger age (OR: 0.97, 95% CI 0.97, 0.97), association with 51–75% of household income (OR: 1.05, 95% CI 1.01, 1.10), previous history of coronary artery bypass grafting (OR: 1.54, 95% CI 1.49, 1.61), myocardial infarction (OR: 1.28, 95% CI 1.24, 1.32), hypertension (OR: 1.11, 95% CI 1.07, 1.14), dyslipidemia (OR: 1.07, 95% CI 1.04, 1.10), and history of cerebrovascular events (OR: 1.18, 95% CI 1.11, 1.24) are more likely characteristics compared with patients who were not readmitted. Patients who underwent PCI for indications other than ACS at index admission were observed to have a greater likelihood of readmission at 30‐days (OR: 8.26, 95% CI 7.99, 8.54). Reduced likelihood is observed in those with a history of smoking, obesity, chronic kidney disease (stage 1–3), family or personal history of IHD, diabetes mellitus, atrial fibrillation, anaemia, peripheral vascular disease and valvular heart disease or history of heart failure prior to index PCI (Supplementary Figure 2).

### 3.4 Comparisons of patients readmitted with angina versus non‐specific chest pain

Compared with patients readmitted within 30 days for nonspecific chest pain, patients who were readmitted with a diagnosis of angina within 30 days were older, more likely to be male, to associate with the 0–25% of household income, have a smoking history, family or personal history of IHD, chronic kidney disease (stage 1–3), dyslipidemia, history of heart failure or valvular heart disease, peripheral vascular disease, anaemia, atrial fibrillation, previous CABG and acute coronary syndrome on admission for index PCI (Supplementary Table 3). Patients with angina readmissions within 30‐days had a higher inpatient mortality rate (Supplementary Table 1). Total charges were greater in those readmitted with angina with longer average duration of admission compared with non‐specific chest pain.

### 3.5 Duration of admission and costs

Patients readmitted with angina or non‐specific chest pain had a shorter duration of readmission hospital stay (mean: 2.3, 95% CI 2.37, 2.42) compared with those readmitted for other causes within a 30‐day period (mean: 5.18 days, 95% CI 5.16, 1.21) (*P* < 0.005). Angina or non‐specific chest pain readmissions were also associated with lower hospitalization costs ($7083) compared with other causes of readmission ($11 642) (*P* < 0.005) (Supplementary Table 2).

## 4 DISCUSSION

We have assessed unplanned readmission with a primary diagnosis of angina or non‐specific chest pain within 30‐days of PCI in a large, national database. We have found that early readmission with angina or non‐specific chest pain after PCI is uncommon. Only 1.5% of patients treated with PCI were readmitted with angina or non‐specific chest pain and affected patients had more cardiovascular risk factors and history of previous IHD. Nonetheless, since many patients undergo PCI, a readmission rate of 1–2% within 30 days equates to a considerable number of patients. Readmission after PCI was associated with an appreciable cost. Mortality during readmission is low in keeping with the coded diagnoses.

### 4.1 Causes of angina post‐PCI

PCI is indicated for patients with anginal symptoms despite guideline‐directed medical therapy to relieve symptoms of angina and may improve prognosis.^9^, ^10^ However, although PCI is routinely successful, angina may persist.^11^ The causes of persisting or recurrent angina include incomplete revascularization, complications of PCI for example, side‐branch loss, or unusually, unsuccessful PCI. A further issue may be the underutilization of available secondary preventative therapy combinations, which may prevent the requirement to progress to invasive management in chronic coronary syndromes.^12^ An under‐recognized problem is ischemia and no obstructive coronary artery disease (INOCA). This group of disorders includes microvascular angina, vasospastic angina or mixed microvascular/vasospastic angina, in the absence of obstructive (≥50% diameter stenosis) or flow‐limiting (fractional flow reserve ≤0.80; non‐hyperemic pressure ratio ≤ 0.89) CAD.^13^ Patients with INOCA have a burden of anginal symptoms and typically poorer quality of life compared to patients with obstructive CAD.^14^ Microvascular angina may be associated with obstructive CAD (Type 3 microvascular angina), or, alternatively, CAD may be falsely classified as obstructive when in fact the primary cause of angina is microvascular disease. Our analysis does not provide information on the etiology of the chest pain in this cohort out with diagnosis code however, it is observed that index acute coronary syndrome was significantly less common in angina and non‐specific chest pain readmissions at 30‐days compared with index population within this cohort. A limitation to this estimate is that readmissions from recurrent myocardial infarction are more likely to occur within an acute coronary syndrome subset rather than readmission with non‐specific or angina pain. Furthermore, identification of culprit arteries may be less clear in patients undergoing PCI for non‐ACS indications so as not, to provide symptomatic benefit for patients with angina, and therefore cause proportionately more readmissions. Further studies to investigate etiology within patients readmitted with chest pain including the success of revascularization, residual coronary disease burden and discharge medication are warranted.

Chest pain after PCI may be experienced in 36% to 42% of patients undergoing both elective and emergent PCI.^15^, ^16^, ^17^ It is most commonly described in the first 24 h following PCI but is described as occurring within the first 3 weeks.^16^ In addition to non‐cardiac causes it is important to distinguish patients with benign chest pain from critical chest pain after PCI due to acute stent thrombosis, incomplete revascularization, or disease progression affecting alternative coronary regions. However, risk stratification in these patients is challenging and may be influenced by the presence of persistently elevated cardiac enzymes or electrocardiograph evolution in the absence of new myocardial injury.^18^, ^19^, ^20^ Benign chest pain and patients with stable angina post‐PCI pain in the absence of ACS, pulmonary or upper gastrointestinal pathologies is therefore understandably recorded in up to one third of overall PCI re‐admissions.^21^, ^22^ No standard nomenclature for the clinical phenomenon of chest pain post‐PCI currently exists due to differing opinions of etiology and there are no guidelines for a standardized approach to management.^23^

A further entity may include the psychological burden associated with a diagnosis of non‐specific chest pain and it is estimated that anxiety disorders are prevalent in 30–50% of these patients.^24^ Somatization disorders with chest pain symptoms may influence readmission, particularly in non‐ACS PCI indications.

### 4.2 Healthcare implications of hospital readmission post‐PCI

Readmissions are a significant source of burden both on the patient and the healthcare system, which is often used as a proxy‐marker for quality of care and penalty systems are implemented for providers with greater proportions of readmission.^25^

Patients with chest pain constitute between 0.6 to 2.4% of unplanned presentations to emergency departments and up to one in four admissions to medical and cardiology wards.^26^, ^27^, ^28^, ^29^ In the United Kingdom, this represents a significant burden with non‐ACS chest pain equating to an average of 15.8 and 16.8 bed days per 1000 population for angina and non‐specific chest pain respectively with standalone 30‐day mortalities of 1.5% and 0.7%.^28^ The incidence and demographical distribution of patients readmitted with chest pain syndromes has not previously been explored. Therefore, the burden on health services as well as mortality and major adverse cardiac event (MACE) rate for patients readmitted with chest pain post‐PCI is not clearly defined.

Our study involved a large sample that is likely to be reasonably representative of the US population undergoing PCI. The NRD has been utilized previously in patients with chest pain, which provides precedent for selection in this study.^30^ Local audit and assessment of chest pain readmissions should be encouraged in order to establish local requirement for interventions, which may reduce readmissions with non‐specific chest pain and angina following PCI. This would ensure appropriate utilization of available resources and financial investment dependent on the localized burden of readmissions.

### 4.3 Associations with cardiovascular risk factors: implications for risk stratification

Demographic factors associated with higher likelihood of unplanned readmission in this sample are in keeping with known cardiovascular risk factors. However, smoking in this sample was not associated with increased readmission at 30 days. This is based on index smoking status and it is plausible that this may be subject to the smoking modification and cessation programmes, which are commonplace in the management of patients with coronary disease. Patients with heart failure, valvular heart disease and non‐cardiac vascular disease were observed to be less likely to be re‐admitted at 30‐days. This is in part due to the proportion undergoing PCI for ACS in whom ventricular dysfunction if present will be identified following PCI rather than as a co‐morbidity on index admission and may also be secondary to increased involvement of secondary care outpatient services in their management and treatment planning.

Optimization of modifiable risk factors prior to intervention is performed in surgical patients and the pre‐operative assessment is commonplace in order to improve surgical morbidity and mortality.^31^, ^32^ However, currently there are no similar formalized pathways for patients undergoing PCI which could be implemented in elective angiography patients to improve long‐term outcomes. Median household income was significantly associated with increased likelihood of readmission at 30‐days in patients with angina and those associating with the 76–100% had a lower likelihood of readmission with a primary diagnosis of angina. Chronic kidney disease in this population was not associated with increased likelihood of re‐admission however, the majority of patients included are of mild impairment as would be expected to undergo PCI with contrast. Female gender was more likely for patients readmitted within 30‐days with angina and non‐specific chest pain and it is previously observed that microvascular and vasospastic angina with INOCA or obstructive CAD is more common in female patients.^33^ Male gender was observed in the majority of all groups in keeping with gender as a known risk factor of cardiovascular disease. Anaemia in this dataset is associated with increased readmission with a primary diagnosis of angina at 30‐days following PCI. Although discharge haemoglobin concentrations are not provided in the database, this may provide an area of potential modification for patients prior to being discharged following PCI.

One evidence‐based example of an intervention to reduce readmissions following PCI is a multimodal strategy as described by Tanguturi et al (2016).^34^ This involved a risk assessment of readmission with the production of patient videos regarding subsequent chest pain or symptoms of heart failure. In addition, a formal clinic review with a cardiology fellow and a computerized alert system for re‐presentations facilitated early cardiologist review. This package of interventions reduced 30‐day hospital readmission from 9.6% to 5.3% over the 4‐year study period.

### 4.4 Limitations

Limitations included the composition of the database from separate yearly data, which prevents multi‐year follow‐up of these patients and excludes those admitted in December from 30‐day follow‐up. On the other hand, a longer follow‐up period for example at 6 months would necessitate the exclusion of the inverse proportion for that year and would have reduced the external validity of the analysis. The database is comprised of inpatient admissions and does not include discharges from emergency care, community data or patients managed in observation areas following PCI. In the United States approximately 7.6 million patients present with chest pain per annum, four out of five will not require admission. A further limitation is the lack of information on completeness of revascularization for included patients or their prescribed medications at the time of discharge. This would be of value in this cohort, particularly where subsequent angina diagnoses are coded on readmission in patients who have undergone PCI for indications other than ACS.

## 5 CONCLUSIONS

Our study provides insights into the prevalence, risk factors and health burden of readmission with angina or non‐specific chest pain following PCI. Secondary prevention measures to reduce cardiovascular risk such as correction of anaemia may help to optimize the clinical status of patients prior to undergoing PCI. PCI performed for an indication other than ACS is associated with a greater likelihood of readmission with angina or non‐specific chest pain at 30‐days within this cohort and further investigation of the etiology within these patients is required.

## CONFLICT OF INTEREST

Professor Colin Berry is employed by the University of Glasgow, which holds consultancy and research agreements for his work with companies that have commercial interests in the diagnosis and treatment of angina. The companies include Abbott Vascular, Astra Zeneca, Boehringer Ingelheim, GSK, HeartFlow, Menarini, Novartis, and Siemens Healthcare. None of the other authors have any potential conflicts of interest.

## Supporting information


**Appendix** **S1**: Supporting information

## Data Availability

The data underlying this article were provided by the Healthcare Cost and Utilization Project under licence. Data will be shared on request to the corresponding author with permission of the Healthcare Cost and Utilization Project

## Appendix

**TABLE A1:** Classification of clinic classification software codes for readmissions causes

Causes of readmission	CCS code	Diagnosis
Respiratory	127	Chronic obstructive pulmonary disease and bronchiectasis
	128	Asthma
	130	Pleurisy, pneumothorax, pulmonary collapse
	131	Respiratory failure, insufficiency and arrest
	132	Lung disease due to external agents
	133	Other lower respiratory disease
	134	Other upper respiratory disease
	221	Respiratory distress syndrome
Infection	1	Tuberculosis
	2	Septicemia
	3	Bacterial infection
	4	Mycoses
	5	HIV infection
	6	Hepatitis
	7	Viral infection
	8	Other infection
	9	Sexually transmitted infection
	76	Meningitis
	77	Encephalitis
	78	Other CNS infection and poliomyelitis
	90	Inflammation or infection of eye
	122	Pneumonia
	123	Influenza
	124	Acute and chronic tonsillitis
	125	Acute bronchitis
	126	Other upper respiratory infections
	129	Aspiration pneumonitis
	135	Intestinal infection
	197	Skin and subcutaneous tissue infections
	201	Infective arthritis and osteomyelitis (except that caused by tuberculosis or sexually transmitted disease)
Bleeding	60	Acute posthaemorrhagic anaemia
	153	Gastrointestinal haemorrhage
	182	Haemorrhage during pregnancy; abrutio placenta; placenta previa
Peripheral vascular disease	114	Peripheral and visceral atherosclerosis
	115	Aortic, peripheral and visceral artery aneurysms
	116	Aortic and peripheral arterial embolism or thrombosis
	117	Other circulatory disease
	118	Phlebitis, thrombophlebitis and thromboembolism
	119	Varicose veins of lower extremities
Genitourinary	159	Urinary tract infection
	160	Calculus of the urinary tract
	161	Other diseases of kidney and ureters
	162	Other diseases of bladder and urethra
	163	Genitourinary symptoms and ill‐defined conditions
	164	Hyperplasia of prostate
	165	Inflammatory conditions of the male genital organs
	166	Other male genital disorders
	170	Prolapse of female genital organs
	175	Other female genital disorders
	215	Genitourinary congenital anomalies
Renal disease	156	Nephritis; nephrosis; renal sclerosis
	157	Acute and unspecified renal failure
	158	Chronic kidney disease
Gastrointestinal	138	Esophageal disorders
	139	Gastroduodenal ulcer (except haemorrhage)
	140	Gastritis and duodenitis
	141	Other disorders of stomach and duodenum
	142	Appendicitis and other appendiceal conditions
	143	Abdominal hernia
	144	Regional enteritis and ulcerative colitis
	145	Intestinal obstruction without hernia
	146	Diverticulosis and diverticulitis
	147	Anal and rectal conditions
	148	Peritonitis and intestinal abscess
	149	Biliary tract disease
	150	Liver disease; alcohol‐related
	151	Other liver diseases
	152	Pancreatic disorders (not diabetes)
	154	Noninfectious gastroenteritis
	155	Other gastrointestinal disorders
	214	Digestive congenital anomalies
	222	Haemolytic jaundice and perinatal jaundice
	250	Nausea and vomiting
	251	Abdominal pain
TIA/stroke	109	Acute cerebrovascular disease
	110	Occlusion of stenosis of precerebral arteries
	111	Other and ill‐defined cerebrovascular disease
	112	Transient cerebral ischemia
	113	Late effects of cerebrovascular disease
Trauma	207	Pathological fracture
	225	Joint disorders and dislocations; trauma‐related
	226	Fracture of neck of femur (hip)
	227	Spinal cord injury
	228	Skull and face fractures
	229	Fracture of upper limb
	230	Fracture of lower limb
	231	Other fractures
	232	Sprains and strains
	233	Intracranial injury
	234	Crushing injury or internal injury
	235	Open wounds of head; neck; and trunk
	236	Open wounds of extremities
	239	Superficial injury; contusion
	244	Other injuries and conditions due to external causes
	260	All (external causes of injury and poisoning)
Endocrine/metabolic	48	Thyroid disorders
	49	Diabetes mellitus without complication
	50	Diabetes mellitus with complication
	51	Other endocrine disorders
	53	Disorders of lipid metabolism
	58	Other nutritional and endocrine/metabolic disorders
	186	Diabetes or abnormal glucose tolerance complicating pregnancy; childbirth; or the puerperium
Neuropsychiatric	650	Adjustment disorders
	651	Anxiety disorders
	652	Attention‐deficit, conduct, and disruptive behavior disorders
	653	Delirium, dementia, and amnestic and other cognitive disorders
	654	Developmental disorders
	655	Disorders usually diagnosed in infancy and childhood or adolescence
	656	Impulse control disorders, NEC
	657	Mood disorders
	658	Personality disorders
	659	Schizophrenia and other psychotic disorders
	660	Alcohol‐related disorders
	661	Substance‐related disorders
	662	Suicide and intentional self‐inflicted injury
	663	Screening and history of mental health and substance abuse codes
	670	Miscellaneous mental health disorders
	79	Parkinson's disease
	80	Multiple sclerosis
	81	Other hereditary and degenerative nervous system conditions
	82	Paralysis
	83	Epilepsy, convulsions
	84	Headache including migraine
	85	Coma, stupor and brain damage
	95	Other nervous system disorders
	216	Nervous system congenital anomalies
	650	Adjustment disorders
	651	Anxiety disorders
	652	Attention‐deficit, conduct, and disruptive behavior disorders
	653	Delirium, dementia, and amnestic and other cognitive disorders
	654	Developmental disorders
	655	Disorders usually diagnosed in infancy and childhood or adolescence
	656	Impulse control disorders
	657	Mood disorders
	658	Personality disorders
	659	Schizophrenia and other psychotic disorders
	660	Alcohol‐related disorders
	661	Substance‐related disorders
	662	Suicide and intentional self‐inflicted injury
	663	Screening and history of mental health and substance abuse codes
	670	Miscellaneous mental health disorders
Hematological/neoplastic	11	Cancer of head and neck
	12	Cancer of esophagus
	13	Cancer of stomach
	14	Cancer of colon
	15	Cancer of rectum and anus
	16	Cancer of liver and intrahepatic bile ducts
	17	Cancer of pancreas
	18	Cancer of other gastrointestinal organs, peritoneum
	19	Cancer of bronchus, lung
	20	Cancer of other respiratory and intrathoracic
	21	Cancer of bone and connective tissue
	22	Melanoma of skin
	23	Other non‐epithelial cancer of skin
	24	Cancer of breast
	25	Cancer of uterus
	26	Cancer of cervix
	27	Cancer of ovary
	28	Cancer of other female genital organs
	29	Cancer of prostate
	30	Cancer of testis
	31	Cancer of other male genital organs
	32	Cancer of bladder
	33	Cancer of kidney and renal pelvis
	34	Cancer of other urinary organs
	35	Cancer of brain and nervous system
	36	Cancer of thyroid
	37	Hodgkin's disease
	38	Non‐Hodgkin's lymphoma
	39	Leukemias
	40	Multiple myeloma
	41	Cancer, other and unspecified primary
	42	Secondary malignancies
	43	Malignant neoplasm without specification of site
	44	Neoplasm of unspecified nature or uncertain behavior
	46	Benign neoplasm of uterus
	47	Other and unspecified benign neoplasm
	59	Deficiency and other anaemias
	61	Sickle cell anaemia
	62	Coagulation and haemorrhagic disorders
	63	Disease of white blood cells
	64	Other hematologic conditions
Rheumatology problem	54	Gout and other crystal arthropathies
Opthalmology problem	86	Cataract
	87	Retinal detachment defects, vascular occlusion and retinopathy
	88	Glaucoma
	89	Blindness and vision defects
	91	Other eye disorders
ENT problem	92	Otitis media and related conditions
	93	Conditions associate with dizziness or vertigo
	94	Other ear and sense organ disorder
Non‐specific chest pain	102	Non‐specific chest pain
Oral health problem	136	Disorders of teeth and jaw
	137	Diseases of mouth; excluding dental
Obstetric admission including pregnancy	174	Female infertility
	176	Contraceptive and procreative management
	177	Spontaneous abortion
	178	Induced abortion
	179	Postabortion complication
	180	Ectopic pregnancy
	181	Other complications of pregnancy
	184	Early or threatened labor
	185	Prolonged pregnancy
	187	Malposition; malpresentation
	188	Fetopelvic disproportion; obstruction
	189	Previous C‐section
	190	Fetal distress and abnormal forces of labor
	191	Polyhydramnios and other problems of amniotic cavity
	192	Umbilical cord complication
	193	OB‐related trauma to perineum and vulva
	194	Forceps delivery
	195	Other complications of birth; puerperium affecting management of mother
	196	Other pregnancy and deliver including normal
	218	Liveborn
	219	Short gestation; low birth weight; and fetal growth retardation
	220	Intrauterine hypoxia and birth asphyxia
	223	Birth trauma
	224	Other perinatal conditions
Dermatology problem	198	Other inflammatory condition of skin
	199	Chronic ulcer of skin
	200	Other skin disorders
Poisoning	241	Poisoning by psychotrophic agents
	242	Poisoning by other medication and drugs
	243	Poisoning by nonmedical substances
Syncope	245	Syncope
Other non‐cardiac	10	Immunization and screening for infectious disease
	45	Maintenance chemotherapy, radiotherapy
	52	Nutritional deficiencies
	55	Fluid and electrolyte disorders
	56	Cystic fibrosis
	57	Immunity disorder
	120	Hemorrhoids
	121	Other diseases of veins and lymphatics
	167	Nonmalignant breast conditions
	168	Inflammatory disease of female pelvic organs
	169	Endometriosis
	172	Ovarian cyst
	173	Menopausal disorders
	202	Rheumatoid arthritis and related disease
	203	Osteoarthritis
	204	Other non‐traumatic joint disorders
	205	Spondylosis; intervertebral disc disorders; other back problems
	206	Osteoporosis
	208	Acquired foot deformities
	209	Other acquired deformities
	210	Systemic lupus erythematosus and connective tissue disorders
	211	Other connective tissue disease
	212	Other bone disease and musculoskeletal deformities
	217	Other congenital anomalies
	237	Complication of device; implant or graft
	238	Complications of surgical procedure or medical care
	240	Burns
	246	Fever of unknown origin
	247	Lymphadenitis
	248	Gangrene
	252	Malaise and fatigue
	253	Allergic reactions
	254	Rehabilitation care; fitting of prostheses; and adjustment of devices
	255	Administrative/social admission
	256	Medical examination/evaluation
	257	Other aftercare
	258	Other screening for suspected conditions (not mental disorders or infectious disease)
	259	Residual codes; unclassified
Heart failure	108	Congestive heart failure non‐hypertensive
Arrhythmia	106	Cardiac dysrhythmias
	107	Cardiac arrest and ventricular fibrillation
Conduction disorder	105	Conduction disorders
Valve disorders	96	Heart valve disorder
Hyper/hypotension	98	Essential hypertension
	99	Hypertension with complications and secondary hypertension
	183	Hypertension complicating pregnancy; childbirth and the puerperium
	249	Shock
Pericarditis	97	Peri‐, endo‐ and myocarditis, cardiomyopathy
Coronary artery disease including angina	101	Coronary atherosclerosis and other heart disease includes angina
Acute myocardial infarction	100	Acute myocardial infarction
Others (cardiac)	103	Pulmonary heart disease
	104	Other and ill‐defined heart disease
	213	Cardiac and circulatory congenital anomalies
