# Supplementary material for: Percutaneous coronary intervention and 30‐day unplanned readmission with chest pain in the United States (Nationwide Readmissions Database)
Source: Clin Cardiol. 2021 Feb 16;44(3):291–306. doi: 10.1002/clc.23543 (PMC7943906; doi:10.1002/clc.23543)
Supplement: Supplementary file 1 — Appendix S1: Supporting information [file CLC-44-291-s001.docx]

## Supplementary Information

## Supplementary Table 1: All-cause inpatient mortality at 30-days in patients re-admitted with chest pain compared with other causes.

|  | Mortality during readmission within 30-days |  |
| --- | --- | --- |
| Other causes | 3.80% | - |
| Combined Angina and Non-specific chest pain | 0.35% | <0.0005 |
| Angina | 0.64% | <0.0005 |
| Non-specific chest pain | 0.09% | <0.0005 |

P-value represents the comparison of inpatient charges in the chest pain groups vs. ‘other causes’.

## Supplementary Table 2: Total inpatient charges of patients readmitted within 30-days following PCI.

|  | Total Charges During Readmission within 30 days ($) | P Value |
| --- | --- | --- |
| Other causes | $11,642 | - |
| Combined Angina and Non-specific chest pain | $7,083 | <0.0005 |
| Angina | $9,368 | <0.0005 |
| Non-specific chest pain | $5,064 | <0.0005 |

P-value represents the comparison of inpatient charges in the chest pain groups vs. ‘other causes’.

## Supplementary Table 3: Relative risk of demographics and characteristics, inpatient mortality and total charges for patients readmitted within 30-days with non-specific chest pain versus angina (>1 favours NSCP; <1 favours Angina).

|  | | Angina | NSCP |  |
| --- | --- | --- | --- | --- |
| Variable | | Unplanned readmission | Unplanned readmission | Mean Difference/Relative Risk |
|  | | (n = 19,183) | (n = 21,714) | (95% CI) |
| Age (year) | | 62.8 | 61.6 | 1.25 (0.99, 1.50) |
| Female | | 38.1% | 43.5% | 1.13 (1.10, 1.15) |
| Mean length of stay (days) | | 3.02 | 1.84 | 1.17 (1.12, 1.23) |
| Quartile of median household income | |  |  |  |
|  | 0-25th | 33.5% | 32.4% | 0.97 (0.95, 0.99) |
|  | 26th-50th | 25.9% | 26.3% | 1.01 (0.99, 1.03) |
|  | 51st-75th | 22.0% | 23.5% | 1.05 (1.02, 1.07) |
|  | 76th-100th | 18.5% | 17.9% | 0.98 (0.95, 1.00) |
| Smoker | | 37.9% | 36.3% | 0.97 (0.94, 0.99) |
| Obesity | | 13.8% | 13.4% | 0.98 (0.95, 1.01) |
| Chronic kidney disease | | 14.8% | 12.6% | 0.91 (0.88, 0.94) |
| Family history of IHD | | 8.6% | 7.5% | 0.93 (0.89, 0.96) |
| Personal history of IHD | | 98.2% | 92.5% | 0.36 (0.32, 0.39) |
| Previous MI | | 24.1% | 24.1% | 1.00 (0.98, 1.02) |
| Dyslipidemia | | 73.6% | 70.5% | 0.92 (0.90, 0.94) |
| Hypertension | | 77.6% | 76.4% | 0.96 (0.93, 0.99) |
| Diabetes Mellitus | | 39.8% | 39.1% | 0.99 (0.97, 1.01) |
| Heart Failure | | 1.4% | 0.4% | 0.59 (0.56, 0.63) |
| Valvular Heart Disease | | 0.5% | 0.1% | 0.56 (0.52, 0.61) |
| History of Stroke/TIA | | 6.2% | 6.7% | 1.04 (1.00, 1.09) |
| Peripheral Vascular Disease | | 10.5% | 7.8% | 0.85 (0.83, 0.88) |
| Anemia | | 13.7% | 11.2% | 0.89 (0.86, 0.91) |
| Atrial Fibrillation | | 11.6% | 8.3% | 0.83 (0.81, 0.86) |
| Previous CABG | | 15.8% | 14.3% | 0.94 (0.92, 0.97) |
| Non-ACS PCI on index admission | | 43.4% | 79.2% | 2.17 (2.13, 2.21) |
| ACS PCI on index admission | | 56.6% | 20.8% | 0.46, (0.45, 0.47) |
|  | STEMI | 11.1% | 8.0% | 0.83 (0.81, 0.86) |
|  | NSTEMI/Unstable Angina | 49.0% | 12.9% | 0.44 (0.43, 0.45) |
| Total Charges during readmission | | $9,368 | $5,064 | $4,405 (4118, 4491) |
| Died during readmission within 30-days | | 0.6% | 0.1% | 0.54 (0.51, 0.58) |

## Supplementary Figure 1: Demographic and clinical characteristics associated with the likelihood of readmission at 30-days with angina or non-specific chest pain following PCI. *(Alternative to Table 2)*


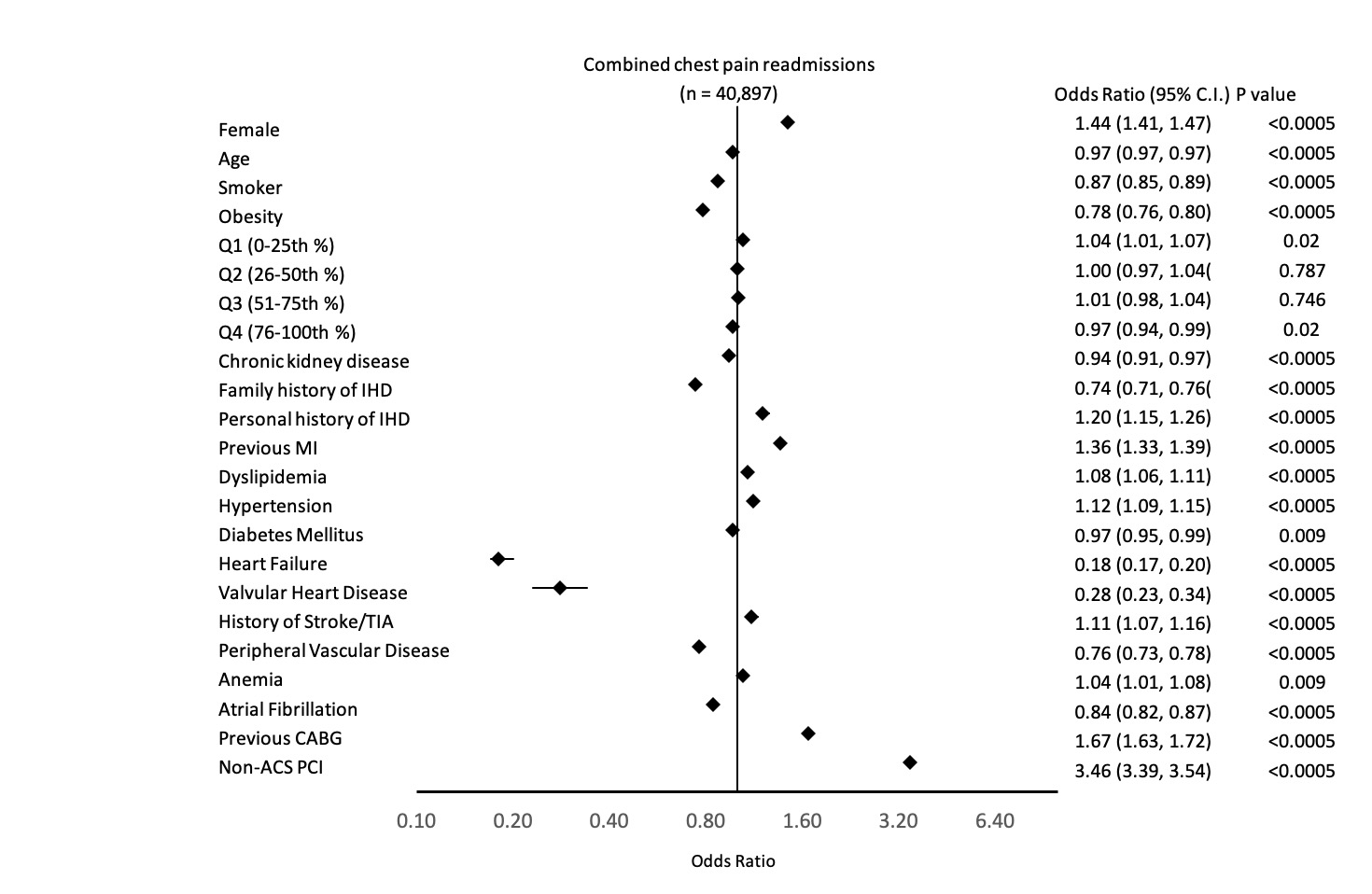


## Supplementary Figure 2: Demographic and clinical characteristics associated with the likelihood of readmission at 30-days with non-specific chest pain following PCI*.*

##
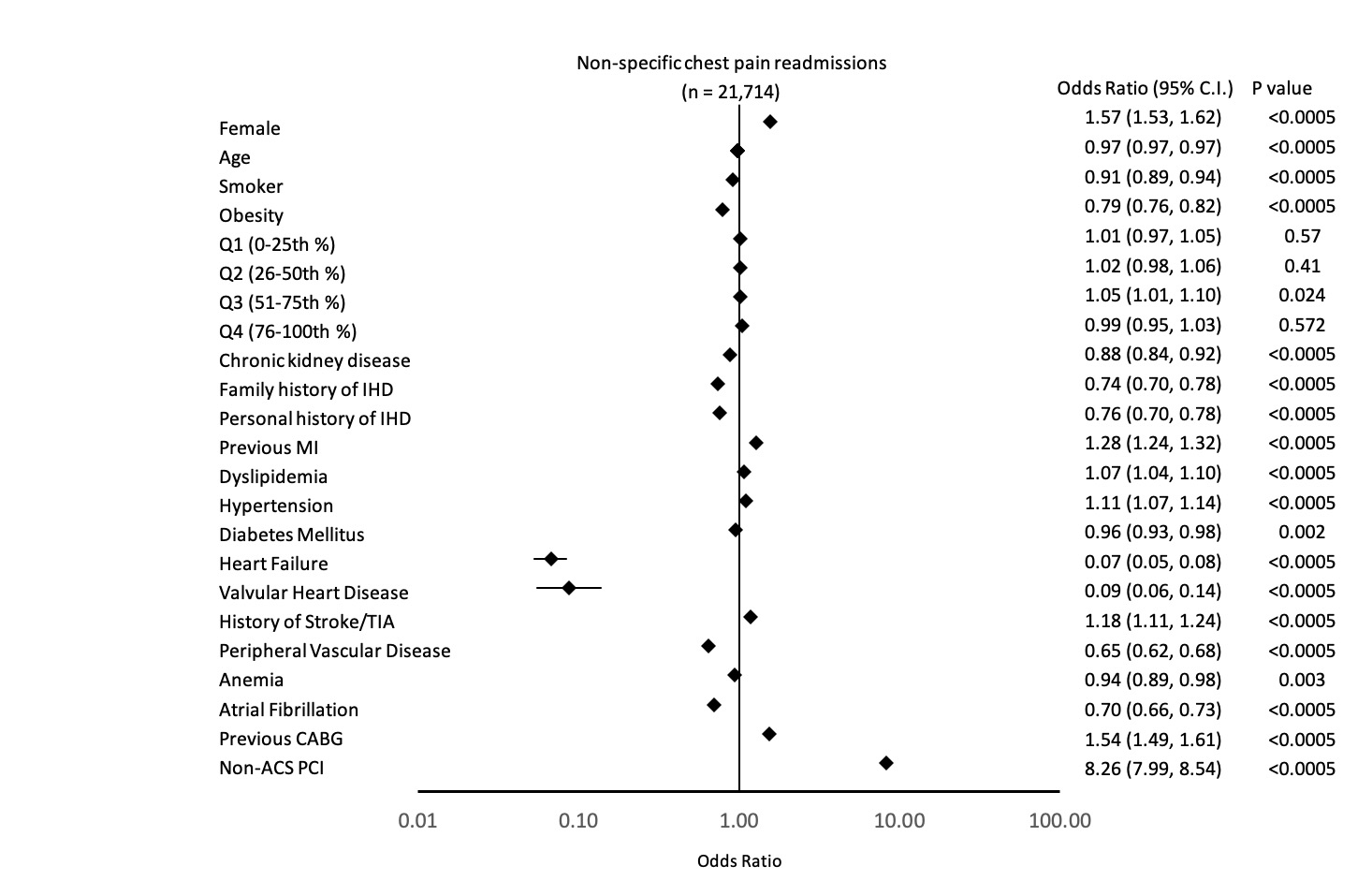


##
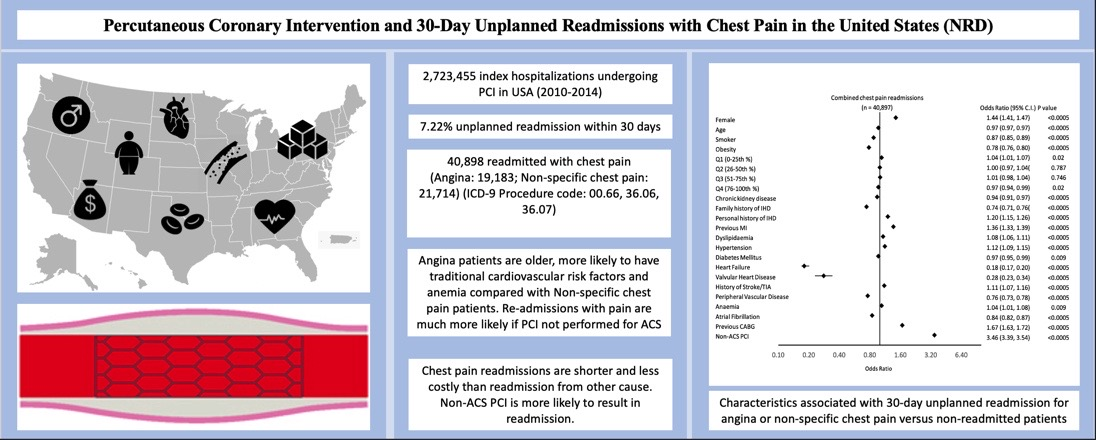
Supplementary Figure 3: Graphical Abstract
